# Alginate based antimicrobial hydrogels formed by integrating Diels–Alder “click chemistry” and the thiol–ene reaction

**DOI:** 10.1039/c8ra00668g

**Published:** 2018-03-21

**Authors:** Gang Wang, Jiehua Zhu, Xiaofeng Chen, Hua Dong, Qingtao Li, Lei Zeng, Xiaodong Cao

**Affiliations:** Department of Biomedical Engineering, School of Materials Science and Engineering, South China University of Technology Guangzhou 510641 PR China caoxd@scut.edu.cn +86-20-22236066; National Engineering Research Center for Tissue Restoration and Reconstruction Guangzhou 510006 PR China; Key Laboratory of Biomedical Materials and Engineering, Ministry of Education, South China University of Technology Guangzhou 510006 PR China; School of Medicine, South China University of Technology Guangzhou 510641 PR China

## Abstract

In recent years medical devices manufacturers have been looking for antimicrobial coatings which are biocompatible and non-toxic for a wide range of medical devices. The demand for these antimicrobial coatings has increased significantly, owing to the increased incidence of hospital-associated infections (HAIs). Hydrogels have been widely used in biomedical applications due to their hydrophilicity, biodegradability, non-toxicity and biocompatibility. In this work, sodium alginate (SA) based antibacterial hydrogels SA/PEG–HHC10 were designed and prepared by combining Diels–Alder (DA) click chemistry and the thiol–ene reaction. The hydrogels were first prepared using DA click chemistry with good mechanical strength, then the cysteine-terminated antimicrobial peptide HHC10–CYS (HHC10) was grafted into the hydrogel by the thiol–ene reaction between the oxy-norbornene group and the thiol group. The results showed that the antimicrobial hydrogels had a strong antibacterial property and good biocompatibility. Therefore, the antimicrobial hydrogels have significant potential application as coatings for implantable medical devices.

## Abstract 2

In recent years medical devices manufacturers have been looking for antimicrobial coatings which are biocompatible and non-toxic for a wide range of medical devices.

## Graphical abstract

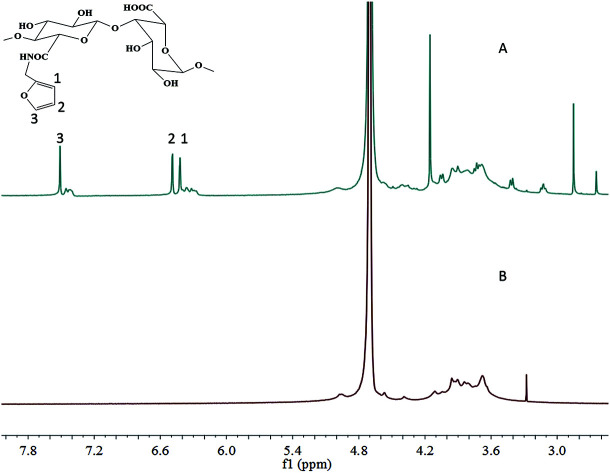

## Introduction

Bacteria can easily adhere and gather on the surface of implantable medical devices and cause bacterial infection.^1^ Bacterial infection may induce inflammation and complications, and in the worst case, even cause implant failure.^2–5^ Once the failure has occurred, the implant needs to be removed and replaced with a new one, significantly increasing the patients' pain and the costs of hospitalization.^6–9^ However, conventional antibiotic treatment often fails due to the low level of antibiotic at the site of infection. Furthermore, over prescription and misuse of conventional antibiotics have led to escalating drug-resistance associated with various bacteria. Therefore, there is a pressing need to develop new antimicrobials and relevant formulations to address these issues.

Hydrogels are three-dimensional networks which are formed by vastly hydrophilic macromolecules.^10^ Due to the unique properties of hydrogels, such as extracellular matrix-like water content, adequate viscoelastic, and desirable biocompatibility, they make excellent candidates for implantable medical device coatings.^11^ As coatings for implantable medical devices, the hydrogels not only need the above mentioned performance characteristics, but more importantly, good antimicrobial properties to ensure the success rate of implanted devices.^11–16^

For this purpose, the common strategies are blending or grafting antibacterial particles or groups into hydrogels.^15^ Employing hydrogels as supports to load the antibacterial particles (typically gold or silver), antibiotics or antimicrobial agents was the first methodology used to obtain antimicrobial hydrogels. However, the application of this approach is limited due to the cytotoxicity of nanoparticles and the short-term antimicrobial activity.^17–19^ The second methodology is chemical grafting of antimicrobial agents onto hydrogels. This approach can enhance the antimicrobial efficacy and specificity, reduce their cytotoxicity, and prolong their biostability and biocompatibility.^20^

Due to widespread resistance to conventional antibiotics, there is increasing interest in developing novel antimicrobials as an alternative antibacterial therapy. The broad-spectrum bactericidal activity of antimicrobial peptides (AMPs) makes them a promising candidate for therapeutic use. AMPs are small molecules that form an important part of the innate immune system. These small molecules are composed of 10–50 amino-acid residues and they show activities against a wide variety of bacterial, viral, fungal and protozoan infections.^21,22^ So far, several combinations of surfaces modified with AMPs as the active antibiotic have been reported.^23–25^ However, it is still a challenge to graft AMPs into biomedical hydrogels with good mechanical strength, strong antibacterial properties and good biocompatibility.

Diels–Alder (DA) click chemistry, a selective [4 + 2] cycloaddition between a diene and a dienophile, can be used as a chemical cross-linking reaction for hydrogels, in particular due to its moderate reaction conditions, thermal reversibility, high efficiency and not requiring a catalyst.^26–30^ Therefore, the hydrogels formed by the DA reaction have played a significant role in biomedical fields.^31–35^ In our previous studies,^36–40^ we developed multifunctional hydrogels with elasticity, injectability, spatiotemporal patterning and tissue adhesive abilities based on DA click chemistry for cartilage tissue repair. In addition, the cyclohexene derivative (oxy-norbornene group) formed by DA click chemistry between furyl and maleimide can go on to efficiently react with the thiol group by the thiol–ene reaction,^41–43^ which can be used to further functionalize hydrogels. For instance, through integrating DA click chemistry and the thiol–ene reaction, we successfully introduced a RGDSC peptide with thiol groups into DA-based hydrogels at a specific time and space.^40^

In this work, DA click chemistry was selected to fabricate a hydrogel with good mechanical properties between a furyl-modified sodium alginate and a bimaleimide functional PEG molecule. Then, the thiol-functionalized antimicrobial peptide HHC10 (HHC10–CYS) with a high activity against multidrug resistant pathogens was selected to react in conjunction with the oxy-norbornene group of the hydrogel. This kind of antimicrobial hydrogel, integrated by DA click chemistry and the thiol–ene reaction, with good mechanical, strong antibacterial properties and desirable biocompatibility has significant potential application for use as a coating for implantable medical devices.

## Materials and methods

### Materials

2-Hydroxy-4-(2-hydroxyethoxy)-2-methylpropiophenone (I2959) and sodium alginate (SA, CP) were purchased from Aladdin. 2-Morpholinoethane sulfonic acid (MES), 4-(4,6-dimethoxy-1,3,5-triazin-2-yl)-4-methylmorpholiniumchloride (DMTMM, CP) and furylamine (furan) were purchased from Sigma-Aldrich (Guangzhou, China). Dimaleimide poly(ethylene glycol) (mal–PEG–mal) (*M*_w_ = 2 × 10^3^) was purchased from Shanghai Sunway Pharmaceutical Technology Co., Ltd (Shanghai, China). HHC10–CYS antimicrobial peptide (H-ckrwwkwirw-NH_2_, 95%, water soluble) was purchased from Hefei peptide Biotechnology Co., Ltd. All other reagents and solvents were of analytical grade and used as received.

## Methods

### Synthesis of SA-furan

**Scheme 1:**
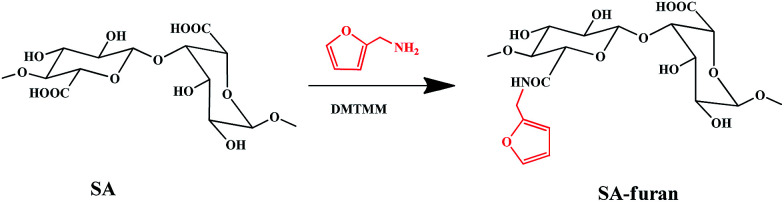
Synthesis steps of SA-furan.

SA-furan was obtained by amidation between the carboxyl groups of SA and the amine groups of furan, as shown in Scheme 1. Briefly, SA (1 g, 5.05 mmol carboxyl groups) was dissolved in 250 ml of MES buffer (100 mM), then DMTMM (0.990 g, 2.5 mmol; 1.397 g, 5 mmol; 2.235 g, 7.5 mmol) was added to activate the polysaccharide carboxyl groups. After stirring for 1 h, furan (343 μl, 2.5 mmol (SA-furan-1); 515 μl, 5 mmol (SA-furan-2); 772 μl, 7.5 mmol (SA-furan-3)) was added dropwise and the mixture was stirred at room temperature for 24 h. The reaction products with different degrees of substitution (DS) were dialyzed against distilled water for 5 days (*M*_w_ cut-off 3500 Da). Finally, the water was removed by lyophilization to obtain SA-furan as a white floccule.

### The formation of SA/PEG hydrogels

**Scheme 2:**
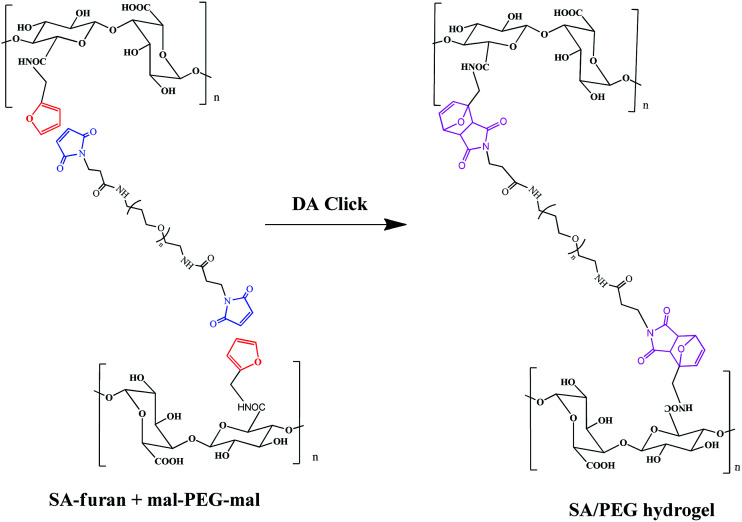
Formation of SA/PEG hydrogels.

Three different degrees of substitution of SA-furan (SA-furan-1, SA-furan-2 and SA-furan-3) were separately dissolved in distilled water with a concentration of 1.5% w/v. The mal–PEG–mal (the molar ratio of furly to maleimide was controlled at 1 : 1) was added into the solutions for 15 min and injected into the mold to form SA/PEG hydrogels (SA/PEG-DS1, SA/PEG-DS2 and SA/PEG-DS3) at 37 °C, separately. Shown in Scheme 2.

### The morphology and gelation time of SA/PEG hydrogels

The morphology of the freeze-dried SA/PEG hydrogels (SA/PEG-DS1, SA/PEG-DS2 and SA/PEG-DS3) was observed using a scanning electron microscope (SEM) (Quanta 200, Netherland FEI). The gelation time of the hydrogels was obtained at 37 °C and 50 °C using a test tube inverting method as described in our previous work.^36^

### Swelling properties of the SA/PEG hydrogels

The SA/PEG hydrogels (SA/PEG-DS1, SA/PEG-DS2 and SA/PEG-DS3) were prepared in a cylindrical mold and were immersed in deionized water at 37 °C for 16 h until the equilibrium of swelling had been reached. The swelling percentage (*Q*_*t*_) at time *t* was calculated using the following equation:*Q*_*t*_ = *W*_*t*_/*W*_0_where *W*_*t*_ and *W*_0_ are the weights of hydrogels at time *t* and time 0, respectively.

The equilibrium swelling ratio (ESR) was calculated using the following equation:
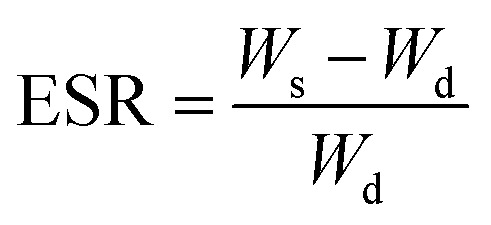
where *W*_s_ and *W*_d_ are the weights of the hydrogels in the equilibrium swelling state and freeze-dried state, respectively.

### Mechanical properties

A dynamic mechanical analyzer (DMA Q800 USA) with unconfined compression mode was used to measure the compressive modulus of the SA/PEG hydrogels (SA/PEG-DS1, SA/PEG-DS2 and SA/PEG-DS3) (cut into cylinders of height 5 mm and diameter 10 mm). The linear ramp force of 1 N min^−1^ up to 18 N was designed to obtain the breaking strength of the hydrogel. The strain from 5 to 10% was used to determine the value of the compressive modulus.

### Synthesis of the antimicrobial hydrogels SA/PEG–HHC10

**Scheme 3:**
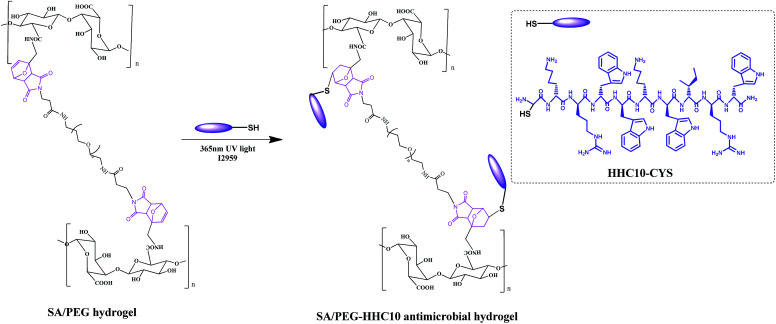
Synthesis of SA/PEG–HHC10 antimicrobial hydrogels.

The synthesis the antimicrobial hydrogels SA/PEG–HHC10 is shown in Scheme 3. Different concentrations of HHC10–CYS antimicrobial peptides were dissolved in MES buffer to prepare a series of concentration solutions (1 mg ml^−1^ (HHC10-I), 2 mg ml^−1^ (HHC10-II), 3 mg ml^−1^ (HHC10-III), 4 mg ml^−1^ (HHC10-IV)). Then, the SA/PEG-DS2 hydrogels with the best mechanical properties in the group were immersed in the HHC10–CYS solution (each sample in 1 ml HHC10–CYS solution) for 24 h, then a photoinitiator I2959 was added and the hydrogels were exposed to 365 nm UV light for 10 min (5 min for each side). These were then washed with PBS buffer to remove the unreacted HHC-10 peptide to achieve SA/PEG–HHC10 antimicrobial hydrogel (SA/PEG–HHC-I, SA/PEG–HHC-II, SA/PEG–HHC-III and SA/PEG–HHC-IV). All the experiments were carried out under aseptic conditions.

### Organic elemental quantitative analysis of SA/PEG–HHC10

SA/PEG–HHC10 hydrogels (SA/PEG–HHC-I, SA/PEG–HHC-II, SA/PEG–HHC-III and SA/PEG–HHC-IV) were freeze-dried and organic elemental quantitative analysis was conducted using an elemental analyzer (vario EL cube, Elementar), the N, S, C elements were measured and the extent of conjugation of HHC-10 antimicrobial peptide to the hydrogel was calculated. The mass of HHC10 in SA/PEG–HHC10 (*m*_H_) can be calculated using the element conservation formula as follows:*m*_H_ = (*m*_Hgel_ × *S*_Hgel_%)/*S*_H_%where *m*_Hgel_ is the mass of the SA/PEG–HHC10 hydrogels, *S*_Hgel_% and *S*_H_% is the S content of the SA/PEG–HHC10 hydrogels and HHC10–CYS, respectively.

### Antibacterial assay of the SA/PEG–HHC10 hydrogel

The SA/PEG–HHC10 hydrogel samples (SA/PEG–HHC-I, SA/PEG–HHC-II, SA/PEG–HHC-III and SA/PEG–HHC-IV) group and blank group (without hydrogel sample) were cultured with 1 ml of the 1 × 10^7^ CFU ml^−1^*Escherichia coli* bacterial stock solution in a 48-well plate for 24 h at 37 °C. After culturing for 24 h, the cultured solutions were diluted 10^4^ times, 10 μl of the 1 ml diluted solution was coated on the agarose culture plate. All agarose culture plates were cultured at 37 °C, with 5% CO_2_ for 10 h to achieve a bacterial colony. The number of CFU in the blank group (*N*_0_) and SA/PEG–HHC10 hydrogel samples groups (*N*_*x*_) was obtained by the flat colony counting method. The sterilization rate (ST_*x*_) was calculated using the following equation:
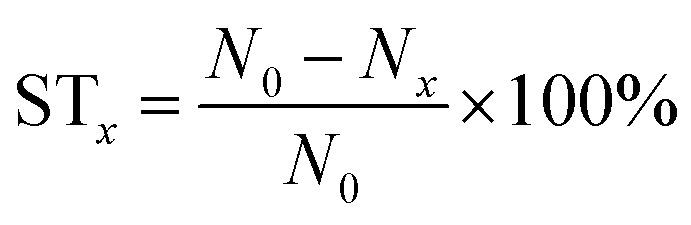


### Biocompatibility assay of SA/PEG–HHC10

Freeze-dried SA/PEG–HHC10 hydrogels (SA/PEG–HHC-I, SA/PEG–HHC-II, SA/PEG–HHC-III and SA/PEG–HHC-IV) were put in a 48-pore plate after radiation sterilization, and the sterile PBS (500 μl) was added, then the extra PBS was removed before adding Dulbecco's modified Eagle's medium (Gibco) (DMEM) with 10% fetal bovine serum, the hydrogels were immersed for 24 h. The human skin fibroblasts (HSF) were seeded onto the surface of the hydrogels with an inoculum density of 2 × 10^4^ CFU cm^−2^. Cell viability was revealed by using the live–dead assay (live–dead assay Kit (Invitrogen)) after culture for 1 and 3 days.

## Results and discussion

### Characterization of SA-furan

**Fig. 1:**
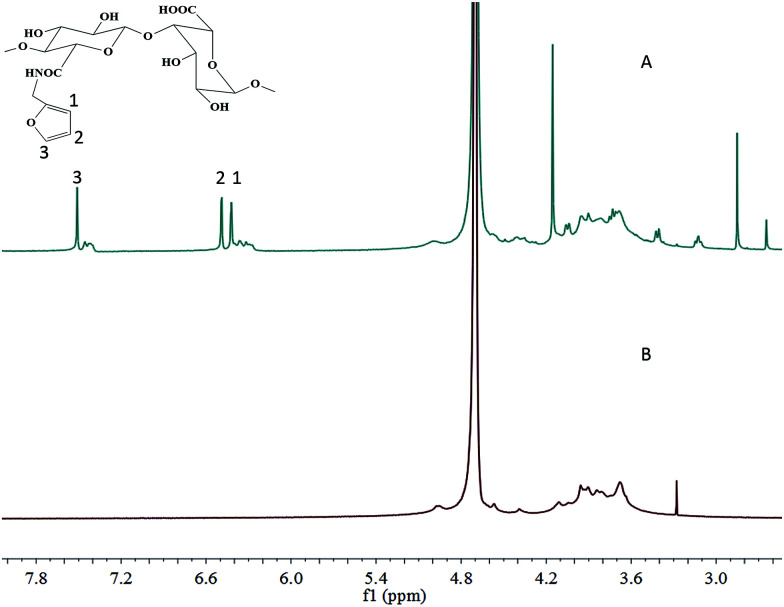
^1^H-NMR spectra in D_2_O (400 MHz) of SA (B) and SA-furan (A) showing that the furan groups were successfully grafted to SA. The peaks at 6.26, 6.46 and 7.51 ppm (furan protons) are indicated by 1, 2 and 3.

SA-furan was obtained by coupling furfurylamine to SA using DMTMM reagent (Scheme 1). The chemical structure of SA-furan was confirmed by ^1^H-NMR (Fig. 1). The peaks at 6.26, 6.46 and 7.51 ppm refer to the protons of furan, these peaks show that the furan group is successfully grafted to SA. The DS of SA-furan was calculated from the C and N content according to the elemental analysis, the calculation of DS was deduced as follows:(*M*_1C_ + DS × *M*_2C_)/(DS × *M*_2N_) = C%/N%where *M*_2N_ is the N mass of furan, *M*_1C_ and *M*_2C_ are the C mass of the SA and furan, respectively.

The DS values for SA-furan were 24.71 ± 1.21%, 35.52 ± 1.21%, and 44.63 ± 1.32% (Table 1) respectively.

**Table 1:** Basic parameters of SA before and after modification

Sample	N content	C content	DS
SA	0.13 ± 0.03	31.13 ± 0.96	—
SA-furan-1	1.35 ± 0.05	33.60 ± 0.37	24.71 ± 1.21%
SA-furan-2	1.75 ± 0.07	34.94 ± 0.47	35.52 ± 1.65%
SA-furan-3	2.19 ± 0.06	34.50 ± 0.50	44.63 ± 1.32%

### Gelation time and morphology of the SA/PEG hydrogels

SA/PEG hydrogels with three different cross-linking densities were prepared (SA/PEG-DS1, SA/PEG-DS2, SA/PEG-DS3). The gelation time of the SA/PEG hydrogels at 37 and 50 °C is shown in Table 2. From Table 2 we can see that with the same DS, the gelation time of 50 °C was shorter than that of 37 °C, which can be explained by the fact that the DA reaction is thermally induced, the reaction rate is faster when the temperature is higher. Compared with 50 °C, the gelation time more sharply decreased with increasing DS at 37 °C, this indicated that the DS was less influenced by the gelation time when the temperature is higher.

**Table 2:** The gelation time of SA/PEG hydrogels with different DS at different temperatures

Sample	37 °C (min)	50 °C (min)
SA/PEG-DS1	110	70
SA/PEG-DS2	100	65
SA/PEG-DS3	90	60

**Table 3:** Organic elemental quantitative analysis of HHC10–CYS

Sample	C content	N content	S content (*S*_H_%)
HHC10–CYS	50.20 ± 0.33	9.21 ± 0.26	2.60 ± 0.32

**Fig. 2:**
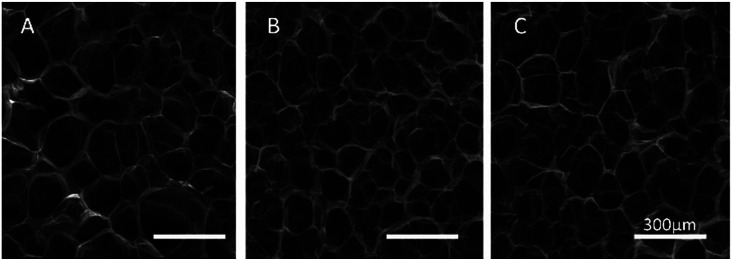
SEM of SA/PEG hydrogels with different DS. SA/PEG-DS1 (A), SA/PEG-DS2 (B), SA/PEG-DS3 (C).

In order to reveal the relationship between the DS and network structure of the SA/PEG hydrogels, the microstructure of the freeze-dried hydrogel was observed using SEM micrographs. As can be seen from A, B and C of Fig. 2, the freeze-dried scaffolds exhibited a uniform pore size and good connectivity. The average pore size of SA/PEG-DS1, SA/PEG-DS2 and SA/PEG-DS3 were 165, 109 and 85 μm, respectively. It can be concluded that the pore size of the hydrogels decreased with an increase of DS, which was caused by the increasing of the crosslinking density of the hydrogels.

### Swelling properties of the SA/PEG hydrogels

**Fig. 3:**
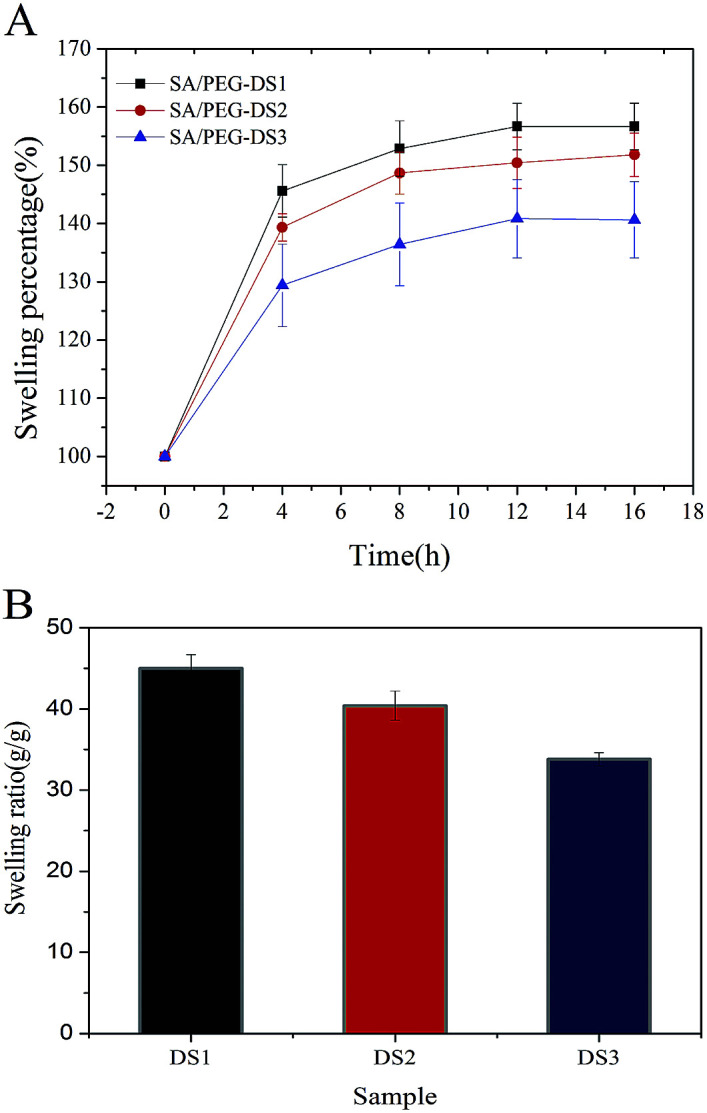
The swelling properties of SA/PEG hydrogels with different DS. (A) The swelling percentage. (B) The equilibrium swelling ratio.

The ESR is an important parameter for the use of hydrogels in biomaterials. It was obtained by recording the water absorption of the hydrogels over time until it reached equilibrium. As shown in Fig. 3, the ESR of SA/PEG-DS1 was 45 g g^−1^, SA/PEG-DS2 was 40 g g^−1^ and SA/PEG-DS1 was 33 g g^−1^. From these results we can see that an increase in the cross-linker concentration led to a decrease in the swelling capacity of the hydrogels. This behavior could have contributed to the increase in the concentration of the hydrophilic cross-linkers within the hydrogel matrix and a tough cross-linked network was obtained.

### Mechanical properties of the SA/PEG hydrogels

**Fig. 4:**
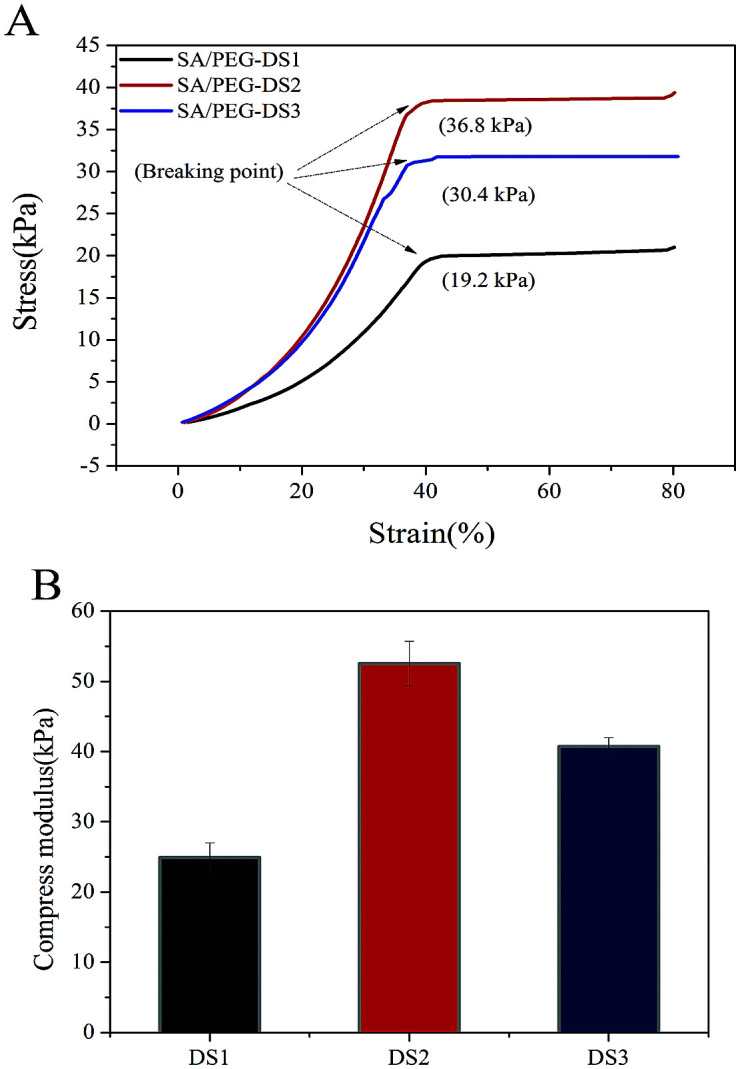
(A) The stress–strain curves of SA/PEG hydrogels by a linear 1 N min^−1^ ramp force loading to test the breakage strength. (B) The compressive modulus of the SA/PEG hydrogels.

Hydrogel samples were tested for their mechanical properties under compression. Typical compressive stress–strain curves of SA/PEG hydrogels with different DS are shown in Fig. 4. With the increasing of DS, the mechanical property first increased and then decreased. The SA/PEG-DS2 had the highest value of stress-at-failure (33.01 ± 4.50 kPa) and compress modulus (52.53 ± 3.16 kPa). This is because the increasing of the crosslinking density of the SA/PEG hydrogels play a major role in the changing of the mechanical properties. When the proportion of flexible molecular chain (PEG) is increased, the increase of flexible segments of the SA/PEG hydrogels, instead of the crosslinking density, plays the main role in the changing of the mechanical properties.

### Mass of HHC10 in SA/PEG–HHC10 hydrogels

The sulfur element (S) content of HHC10–CYS and SA/PEG–HHC10 antibacterial hydrogels were proceeded by organic elemental quantitative analysis. As shown in Table 4, with the increase of soaking concentration, the mass of HHC10 increased firstly and then decreased. The reasons might be the change of hydrogel network size. In the experiment we found that the equilibrium volume increased firstly and then decreased, which is consistent with the mass of HHC10 in the SA/PEG–HHC10 hydrogels (Table 4). The diffusion of peptides depends on the size of the hydrogel network, when the soaking concentration increased to 4 mg ml^−1^, the hydrogels became shrunken, the size of the hydrogel network became smaller than other groups, which caused a decrease in the mass of HHC10 in the SA/PEG–HHC10-IV hydrogel.

**Table 4:** The mass of HHC10 in SA/PEG–HHC10 hydrogels

Sample	*m*_Hgel_ (mg)	*S*_Hgel_%	*m*_H_ (mg)
SA/PEG–HHC10-I	15.88 ± 0.75	0.15	0.91
SA/PEG–HHC10-II	15.69 ± 0.92	0.30	1.8
SA/PEG–HHC10-III	15.33 ± 0.88	0.37	2.2
SA/PEG–HHC10-IV	16.52 ± 0.78	0.24	1.5

### Antibacterial activity of the SA/PEG–HHC10 hydrogels

**Fig. 5:**
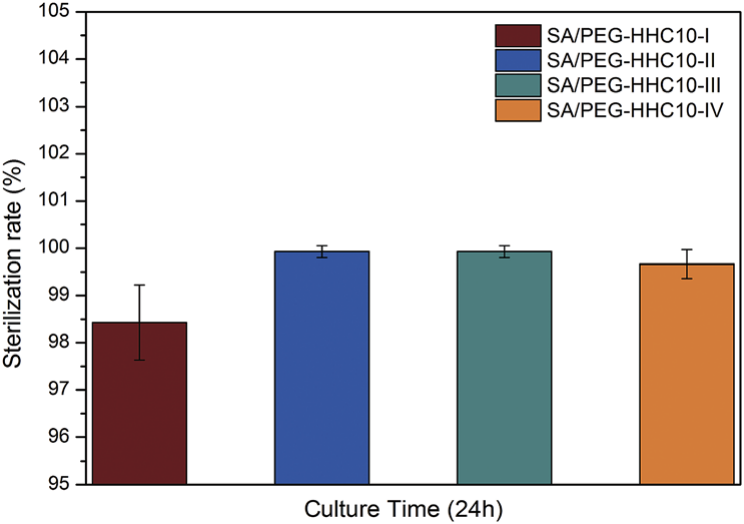
The antibacterial test of SA/PEG–HHC10.

An ideal implantable medical devices coating should possess antimicrobial properties against infection. The antibacterial activity of the SA/PEG–HHC10 hydrogels against *E. coli* is evaluated in Fig. 5. After culture for 24 h, the sterilization rate of SA/PEG–HHC10-I was 98.5%, SA/PEG-HHC10–IV was 99.5% and both SA/PEG–HHC10-II and SA/PEG–HHC10-III were 100%. These results were in accordance with the mass of HHC10 in the SA/PEG–HHC10 hydrogels (Table 4) and show the strong antibacterial activity of the SA/PEG–HHC10 hydrogels.

### Cytocompatibility of the SA/PEG–HHC10 hydrogel

**Fig. 6:**
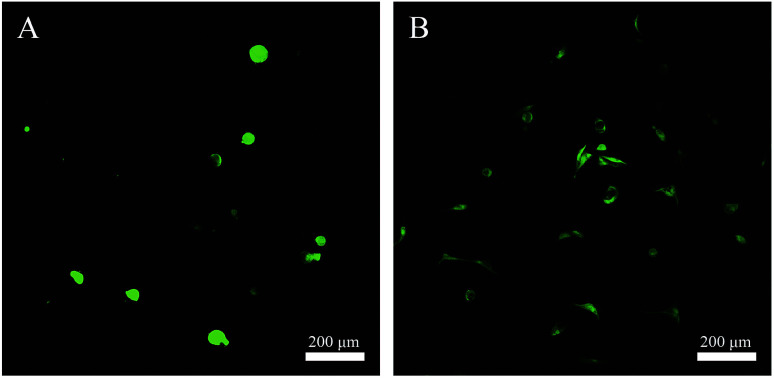
Live–dead assay of HSF cells on SA/PEG–HHC10 hydrogels. Living cells appearing green. The live–dead assay of HSF cells on SA/PEG–HHC10 hydrogels after (A) 1 and (B) 3 days culture.

Cytocompatibility of the SA/PEG–HHC10 hydrogel was assessed using a live-dead assay that stains live cells green (calcein AM) and dead cells red (ethidium homodimer). As shown in Fig. 6(A and B), with the extension of the incubation time, the number of HSF cells on the SA/PEG–HHC10 became more and the morphology of the cells became exclusively a fusiform shape. The results showed that the SA/PEG–HHC10 hydrogel had good cytocompatibility.

## Conclusions

In this work, we used DA click chemistry to form SA/PEG hydrogels with regulated mechanical properties and gelation times. The hydrogel with DS2 has the best mechanical properties in the series of SA/PEG hydrogels, the stress-at-failure and compress modulus were 33.01 ± 4.50 kPa and 52.53 ± 3.16 kPa, respectively. The antimicrobial peptide HHC10 was successfully introduced into the SA/PEG hydrogels by a photoinitiated thiol–ene click reaction between the thiol group and oxy-norbornene group, the sterilization rate reached 100% when a sufficient amount of HHC10 was incorporated. Therefore, the hydrogels SA/PEG–HHC10 had strong antibacterial properties and good biocompatibility. We believe that these antimicrobial hydrogels could be used as coatings for implantable medical devices.

## Conflicts of interest

There are no conflicts to declare.

## Supplementary Material
